# Residual Stress Analysis in Linear Friction Welded Ti17

**DOI:** 10.3390/ma17184507

**Published:** 2024-09-13

**Authors:** Peng He, Yunxin Wu, Tao Zhang, Junlong Jin

**Affiliations:** 1 Light Alloy Research Institute, Central South University, Changsha 410083, China; 213801008@csu.edu.cn; 2 State Key Laboratory of Precision Manufacturing for Extreme Service Performance, Changsha 410083, China; 3 AVIC Manufacturing Technology Institute, Beijing 100024, China; jinjl@avic.com

**Keywords:** Ti17, linear friction welding, residual stress, numerical model, prediction method

## Abstract

Residual stresses with a complex distribution are generated after linear friction welding, which affects the service performance of the weldment. In this work, a numerical model for linear-friction-welded Ti17 (Ti-5Al-2Sn-2Zr-4Mo-4Cr) was developed to investigate the evolution of residual stresses and the effect of welding parameters on residual stresses. Additionally, a method for predicting internal residual stresses was constructed. The results indicate that the residual stresses near the contact interface are largest in the oscillatory direction, peaking at ~661 MPa at 2 mm away from the contact interface. The evolution of stresses is not only related to the inhomogeneous thermal gradient, but also to the forging force. And the stress distribution essentially stabilizes within the duration of the forging force applied. Increasing the amplitude and frequency results in higher peaks of tensile residual stresses and a more concentrated distribution. Conversely, increasing the forging force only reduces the magnitude of the residual stresses. The developed prediction method, based on the similarity of internal residual stress distributions, facilitates the prediction of internal residual stresses using measured surface residual stresses.

## 1. Introduction

Titanium alloys, particularly Ti17 (Ti-5Al-2Sn-2Zr-4Mo-4Cr), are crucial in the aerospace industry due to their high specific strength, wide range of operating temperatures, and excellent corrosion resistance [1]. However, the welding of titanium alloys presents unique challenges, as titanium alloys in the molten stage are prone to react with atmospheric gasses such as nitrogen, oxygen, and hydrogen, resulting in severe embrittlement [2]. Linear friction welding (LFW), as a solid-state welding technique, has attracted significant attention in the manufacture of bladed disks for aero-engines [3]. LFW combines two titanium components through a relative reciprocating motion under axial force [4]. This process retains the titanium components in the solid-state, which means that shielding gas is not required and defects such as porosity and cracks, which may occur in conventional fusion welding, are effectively avoided [3,5].

Nonetheless, unavoidable residual stresses are introduced in the process of LFW due to the inhomogeneous temperature field near the contact interface as well as the large plastic deformation experienced by the titanium components at high temperatures [6]. With the removal of external loads, residual stresses remain in the weldment and maintain self-equilibrium [7]. Residual stresses are a critical factor affecting the service performance of components, such as material strength and fatigue life [8]. Consequently, the precise quantification and analysis of residual stresses are essential in industrial production.

In order to address the above challenges, numerical simulation is frequently employed to investigate residual stresses. Due to the complex thermo-mechanical coupling, LFW is generally divided into two stages—the oscillatory stage (components heating due to relative motion) and the forging stage (components cooling following the cessation of relative motion). However, the existing studies on the numerical simulation of residual stresses in the process of LFW remain limited. Turner et al. [6] developed a two-dimensional model neglecting residual stresses perpendicular to the oscillation direction to study the residual stresses in linear-friction-welded (LFWed) Ti-64. It was found that residual stresses primarily develop during the forging stage, with the distribution and magnitude significantly influenced by the temperature distribution at the end of the oscillatory stage. Nikiforov et al. [9] established a three-dimensional model to study the residual stresses in LFWed Ti-64. The temperature field in the oscillatory stage obtained by the one-dimensional model was introduced into the three-dimensional model to simulate the evolution of stresses obtained during the forging stage. The results indicated that peak tensile residual stresses in the oscillation direction were inversely proportional to the forging force. Fu et al. [10] also developed a three-dimensional model to investigate residual stresses in LFWed Ti-64, taking into account the morphology of the flash. To simplify the friction in the oscillatory stage, the temperature field during the oscillatory stage was considered as a function about the distance from the contact interface. Similarly, the findings demonstrated that the forging force has a significant effect on the magnitude of residual stresses. Inevitably, the parameters of the function regarding the temperature field affected the accuracy of the model. Bühr et al. [11] further considered the axial shortening during the oscillatory stage by applying a heat flux directly at the contact interface and removing the mesh sequentially. This three-dimensional model bypassed the oscillatory stage, thereby improving computational efficiency. Combined with experimental measurements, the model was validated as being reasonable. Additionally, it was found that the effect of friction force on residual stresses is relevant to the variation in the temperature field near the contact interface.

In addition to the numerical simulation, practical measurements are also performed in the study of residual stresses. The contour method and others are commonly employed to obtain internal residual stresses in the weldment [12]. However, these methods are generally destructive and unsuitable for piece-by-piece measurements on titanium components prior to service [13]. Although the X-ray and neutron diffraction methods do not damage titanium components, the information on internal residual stresses is not available [14,15]. Therefore, predicting the internal residual stresses without destroying the titanium components is crucial.

In this work, a three-dimensional modeling approach was developed to analyze the formation of residual stresses during LFW and the effect of welding parameters on residual stresses. Based on simulation results, a shape function of internal residual stresses was obtained, and the relationship between internal and surface residual stresses was derived further. The goal of predicting the internal residual stresses through non-destructive measurements for surface residual stresses is achieved, offering significant advantages for industrial applications.

## 2. Methodology

### 2.1. Experiments

#### 2.1.1. LFW

**Figure 1:**
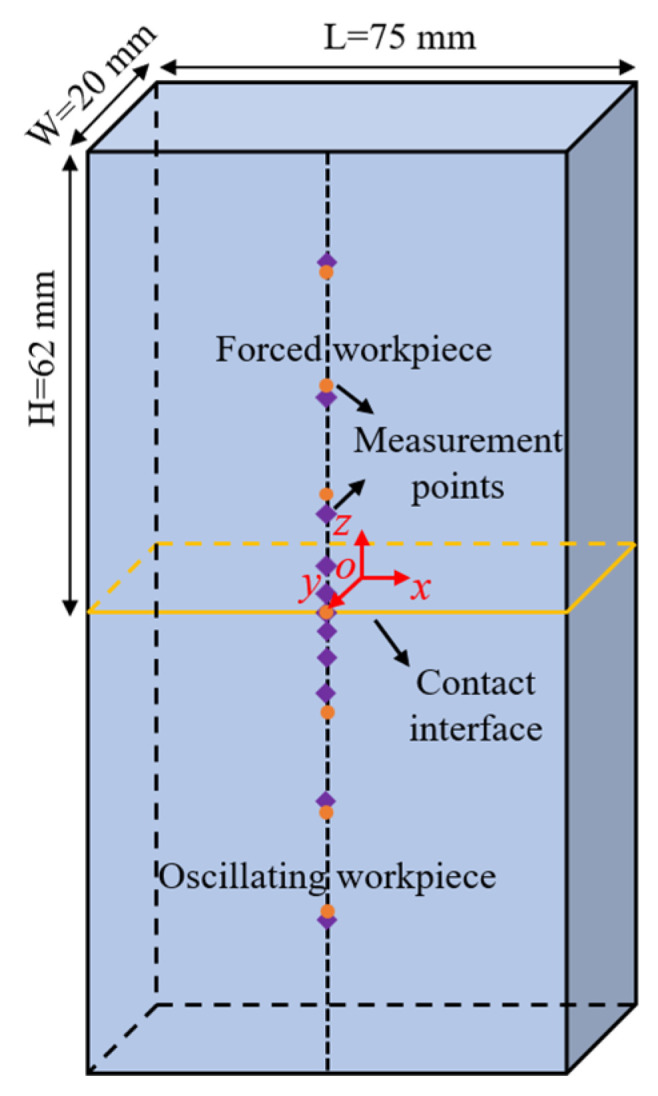
Schematic diagram of the weldment.

The chemical composition of Ti17 (Baomeitai Metal Materials Co., Baoji, Shaanxi, China) as a parent material is shown in Table 1. The parent material machined to dimensions of 75 mm (L), 20 mm (W), and 62 mm (H) was welded using the LFW-60 at the AVIC Manufacturing Technology Research Institute (Beijing, China). The welding parameters employed were as follows: an amplitude of 3 mm, a frequency of 40 Hz, a friction force of 50 MPa, and a forging force of 60 MPa. The oscillatory time was about 4.5 s. The contact interface was L × W, with the direction of relative motion along L, as illustrated in Figure 1.

#### 2.1.2. Residual Stress Measurement

Surface residual stresses were first measured using the X-ray diffraction method in LFWed Ti17. The Proto LXRD Modular Mapping System (Canada) was employed, utilizing CuK_α_ radiation with a voltage of 40 kV, a current of 25 mA, a diffraction angle of 139.69°, a beam diameter of 2 mm, an exposure time of 30 s, and an exposure number of 10. Prior to measurement, the surfaces were cleaned and electrochemically polished to remove oxidized, as well as additional, stress layers. A total of 13 measurement points (purple diamonds) were located 0, 2, 5, 8, 11, 26, and 41 mm from the contact interface, as shown in Figure 1.

Subsequently, the in-plane residual stresses near the surface were measured using the hole-drilling method. According to ASTM E837, the elastic strain at the hole location due to stress relief can be measured by the strain gage rosette, and then the average residual stresses in the depth direction of the drilling are calculated based on the mathematical relationship of linear elasticity theory. The measurement points were polished to remove surface contaminants before applying the strain gage rosette. The holes were drilled using the HK-21B drilling device (Huayun Electromechanical Technology Co., Jinan, Shandong, China) with a drill diameter of 1.5 mm and a hole depth of 2 mm. The uT7110Y static strain indicator (uTekL Co., Wuhan, Hubei, China) was used to obtain the strain results. Each measurement point (orange circle) is spaced 13.5 mm apart, as shown in Figure 1. The calculation relationship is shown below [16]:

(1) $$\left\{\begin{array}{l} \sigma_{\max}=\frac{\varepsilon_{1}+\varepsilon_{3}}{4A}+\frac{1}{4B}\sqrt{\frac{{\left(\varepsilon_{1}-\varepsilon_{3}\right)}^{2}+{\left(\varepsilon_{1}+\varepsilon_{3}-2\varepsilon_{2}\right)}^{2}}{2}}\\ \sigma_{\min}=\frac{\varepsilon_{1}+\varepsilon_{3}}{4A}-\frac{1}{4B}\sqrt{\frac{{\left(\varepsilon_{1}-\varepsilon_{3}\right)}^{2}+{\left(\varepsilon_{1}+\varepsilon_{3}-2\varepsilon_{2}\right)}^{2}}{2}}\\ \tan2\theta =\frac{\varepsilon_{1}+\varepsilon_{3}-2\varepsilon_{2}}{\varepsilon_{3}-\varepsilon_{1}} \end{array}\right.,$$

where *σ*_max_ and *σ*_min_ are the maximum and minimum principal stresses, respectively. *ε*_1_, *ε*_2_, and *ε*_3_ are the strains in the three directions of the strain gage rosette, respectively. *θ* denotes the clockwise angle from the strain grid 1# on the strain gage rosette to the maximum principal stress direction. *A* and *B* represent strain release coefficients. In this paper, *A* = −0.65 and *B* = −1.5.

In the end, the internal residual stresses were obtained using the contour method. The *yoz* plane was cut with a 0.2 mm brass wire on the DK7625P electric discharge machine (Suzhou Sanguang Technology Co., Suzhou, Jiangsu, China), operating at a cutting speed of 1 mm/min. After cutting, the contours of the cutting surfaces were measured using the HEXAGON GLOBAL 575 coordinate measuring machine (Stockholm, Sweden). The contour data were processed by aligning, smoothing, and enveloping, which were then introduced into ABAQUS/standard (ABAQUS 2017) to obtain the residual stresses in the x-direction on the *yoz* plane, as presented in Figure 1.

### 2.2. Numerical Model

According to a previous study, the residual stresses are predominantly influenced by the evolution of temperature during the forging stage, rather than by the stresses generated during the oscillatory stage [6]. Therefore, in this work, LFW was also divided into oscillatory and forging stages for numerical modeling. The final temperature field obtained from the numerical model of the oscillatory stage (oscillatory model) was introduced into the numerical model of the forging stage (forging model) as the initial temperature field for the calculation of residual stresses. This approach ensures the temperature field is mainly concerned in the oscillatory model and the stress field is mainly concerned in the forging model. ABAQUS software was employed for modeling due to the advantage of solving nonlinear problems [17].

**Figure 2:**
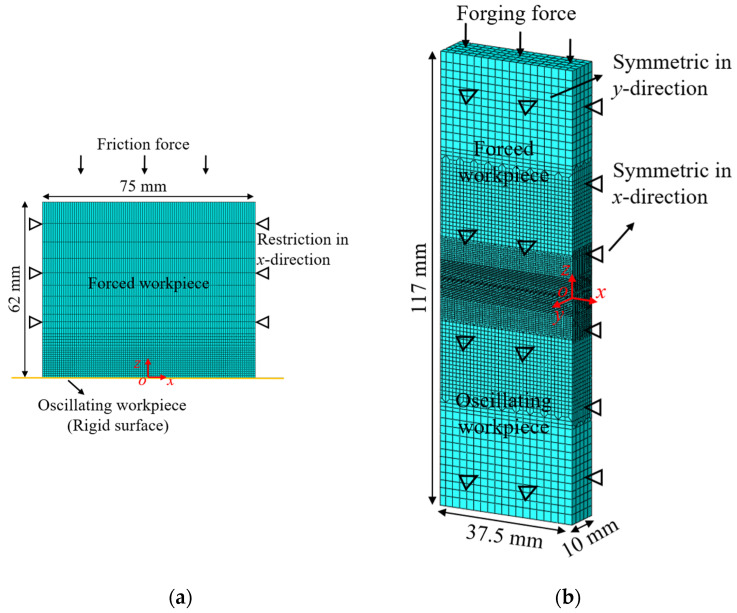
Numerical models of (**a**) oscillatory stage; (**b**) forging stage.

The oscillatory stage was simplified to a two-dimensional model considering that the temperature gradient in the *y*-direction during this stage was not significant [18], as illustrated in Figure 2a. The forced workpiece was composed of CPE4RT quadrilateral elements, with a size of 0.75 mm near the contact interface and gradually increasing to 7.5 mm at the edges, in order to capture the steep temperature gradient near the contact interface. The degrees of freedom in the *x*-direction at the edges of the forced workpiece were restricted. The oscillating workpiece was replaced by a rigid surface [19]. To prevent computational interruptions caused by excessive distortion of the mesh, Arbitrary Lagrangian Eulerian adaptive mesh control was applied. The computation was ceased once the axial shortening reached 3.5 mm. The friction coefficients used were taken from Zhao et al. [19]. This modeling approach offers an advantage over previous methods [9,10,11] by considering the effect of flash generation on the temperature field.

**Figure 3:**
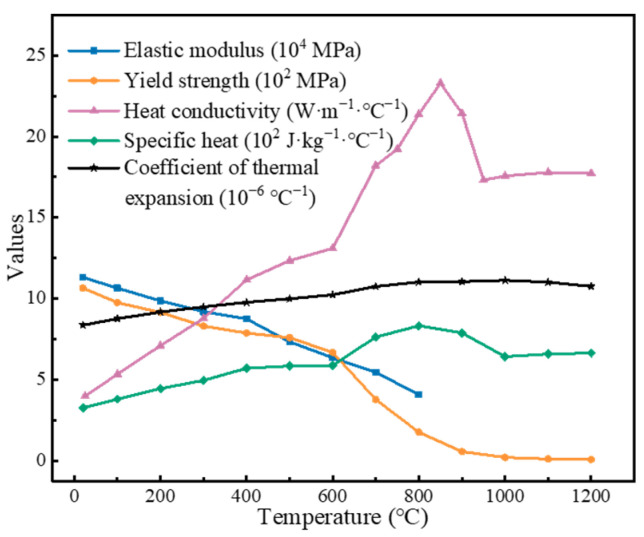
Material properties of Ti17 used in numerical model.

Given the axial shortening that occurs during the oscillatory stage, a three-dimensional model with dimensions of 37.5 × 10 × 117 mm was established based on ABAQUS/Implicit, as shown in Figure 2b. The model represented a quarter of the weldment due to the symmetry of the geometry and boundary conditions about the *xoz* and *yoz* planes. C3D8T hexahedral elements were applied with a size of 0.2 mm near the contact interface and gradually coarsening to 2 mm at the edges. The temperature-dependent Ti17 material properties used are shown in Figure 3. In addition, the mapping of the temperature field from the oscillatory model to the forging model was achieved using the mesh mapping method.

## 3. Results and Discussion

### 3.1. Residual Stresses in LFWed Ti17

**Figure 4:**
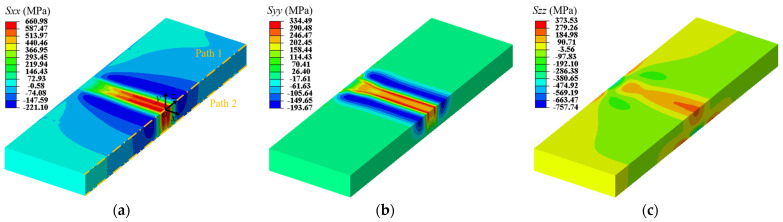
Contours of the residual stresses in LFWed Ti17: (**a**) *S_xx_*; (**b**) *S_yy_*; and (**c**) *S_zz_*.

The contours of residual stresses in the *x*-direction (*S_xx_*), *y*-direction (*S_yy_*), and *z*-direction (*S_zz_*) are shown in Figure 4. It can be clearly seen that the residual stress distribution is notably complex in a narrow zone near the contact interface, where both peak tensile and peak compressive stresses are concentrated. The magnitude of tensile residual stresses is the largest in the *x*-direction and the smallest in the *z*-direction, which is in agreement with previous studies [12,20]. Therefore, *S_xx_* is the main focus of this work.

**Figure 5:**
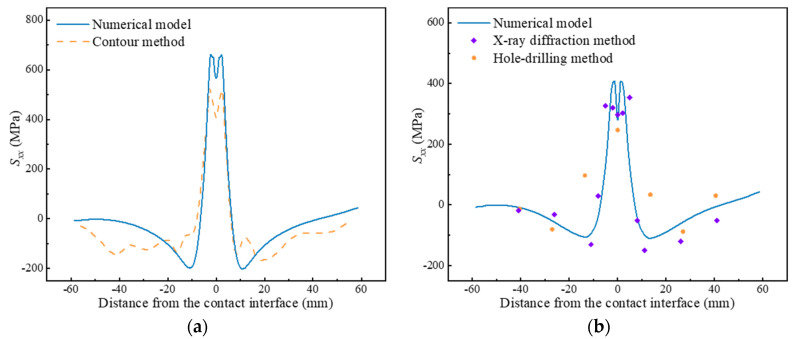
Comparison of results of numerical model and experiments: (**a**) *S_xx_* in LFWed Ti17 along Path 1; (**b**) *S_xx_* in LFWed Ti17 along Path 2.

For a clearer description of the internal residual stresses in LFWed Ti17, Figure 5a plots the *S_xx_* along path 1, which exhibits a bimodal distribution. The tensile stresses are predominantly concentrated in a zone of 12 mm near the contact interface, peak (~661 MPa) at 2 mm away from the contact interface, and valley (~566 MPa) at the contact interface. Beyond a distance of 6 mm from the contact interface, the residual stresses convert from tensile to compressive and approach zero at the edges. The peak compressive stresses appear at about 11 mm away from the contact interface, with a value of ~202 MPa. Figure 5a compares the internal residual stresses obtained from the numerical model with those measured using the contour method, demonstrating good agreement.

Figure 5b plots the surface residual stresses in LFWed Ti17 (*S_xx_* along path 2), illustrating that the distribution of surface residual stresses resembles that of the internal residual stresses, but with a lower peak of ~407 MPa. The results of the X-ray diffraction show good agreement with that of the numerical model, with a numerically lower peak. The XRD diffraction method tends to underestimate the peak when applied to the stress-concentrated workpieces, due to the fact that the average elastic strain within the 2 mm diameter range (beam diameter) is obtained using the XRD diffraction method. However, considering that the residual stresses measured by the hole-drilling method are the average in the range of strain gage rosette dimensions along the hole depth, the difference between the results of the hole-drilling method and those of the numerical model is acceptable. In summary, the experimental results confirm the validity of the numerical model.

### 3.2. Evolution of Stresses

The evolution of stresses during LFW is complex. During the forging stage, the evolution of stresses is induced by strains, which satisfy the following relationship [21]:

(2) $$\Delta \varepsilon =\Delta \varepsilon_{E}+\Delta \varepsilon_{P}+\Delta \varepsilon_{T}+\Delta \varepsilon_{C}+\Delta \varepsilon_{V},$$

where Δ*ε*, Δ*ε_E_*, Δ*ε_P_*, Δ*ε_T_*, Δ*ε_C_*, and Δ*ε_V_* represent the total strain increment, the elastic strain increment, the plastic strain increment, the thermal strain increment, the creep induced strain increment, and the phase transformation induced strain increment, respectively. Since creep is highly time-dependent, Δ*ε_C_* is not taken into account during the forging stage due to the short welding time. Δ*ε_V_* can also be neglected [21]. Therefore, Equation (2) can be simplified to Equation (3):

(3) $$\Delta \varepsilon =\Delta \varepsilon_{E}+\Delta \varepsilon_{P}+\Delta \varepsilon_{T}$$

**Figure 6:**
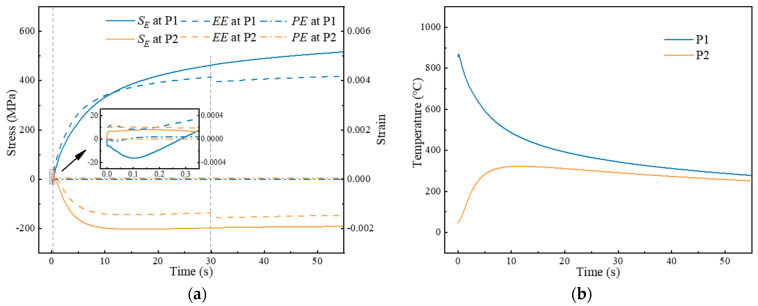
Evolution of (**a**) stresses, elastic strains, and plastic strains in the *x*-direction; (**b**) temperatures at P1 and P2 regarding time.

In order to quantitatively analyze the evolution of stresses during LFW, Figure 6 illustrates the evolution of stresses, strains, and temperatures along Path 1 at the location of peak tensile stress (P1) and the location of peak compressive stress (P2). It should be noted that the stress (*S_E_*), elastic strain (*EE*), and plastic strain (*PE*) are all extracted in the *x*-direction. At the origin of the forging stage, a sharp increase in both compressive stress and strains is observed at P1, attributed to the compressive plastic deformation caused by the application of the forging force. The absence of plastic deformation at P2 is because of the high yield strength at a low temperature of 47 °C, which suggests that the tensile stresses observed here may be due to the self-equilibrium of stresses.

Within 0 to 0.12 s, the temperature at P1 increases from 854 °C to 868 °C due to heat transfer from the contact interface. Thermal expansion occurs at P1 with squeezing by the surrounding material, resulting in a gradual increase in compressive *S_E_* at P1 to 16 MPa. Considering that the yield strength decreases with increasing temperature, the *EE* at P1 decreases to 1.65 × 10^−4^, while the *PE* at P1 reaches 2.81 × 10^−5^. Accordingly, P2 experiences tension, leading to an increase in tensile *S_E_* at P2 to 8 MPa and a corresponding *EE* at P2 of 2.05 × 10^−4^.

Within 0.12 to 30 s, the temperature at P1 decreases to 344 °C. The contraction due to cooling occurs, resulting in *S_E_* at P1 converting from compressive to tensile, gradually increasing to 463 MPa. The *EE* at P1 gradually increases to 4.15 × 10^−3^, while the *PE* at P1 stabilizes at 3.69 × 10^−5^. This stabilization occurs due to the fact that plastic deformation no longer occurs when *S_E_* at P1 is unable to exceed the increasing yield strength with decreasing temperature. At P2, the temperature first rises to 323 °C and then falls to 291 °C, resulting in expansion and then contraction. Therefore, it is observed that the compressive *S_E_* at P2 first increases to 203 MPa and then decreases to 198 MPa, similar to the evolution of *EE* at P2.

After 30 s, the growth rate of *EE* at P1 and P2 slows down due to the similar cooling rates of P1 and P2, leading to a stabilization of the stress distribution in LFWed Ti17. The gradual increases in the magnitude of *S_E_* at P1 and P2 can be attributed to the temperature-dependent elastic modulus. It is important to note that the fluctuation of *EE* at P1 and P2 that occurs at 30 s is due to the removal of the forging force.

### 3.3. Effect of Welding Parameters

**Figure 7:**
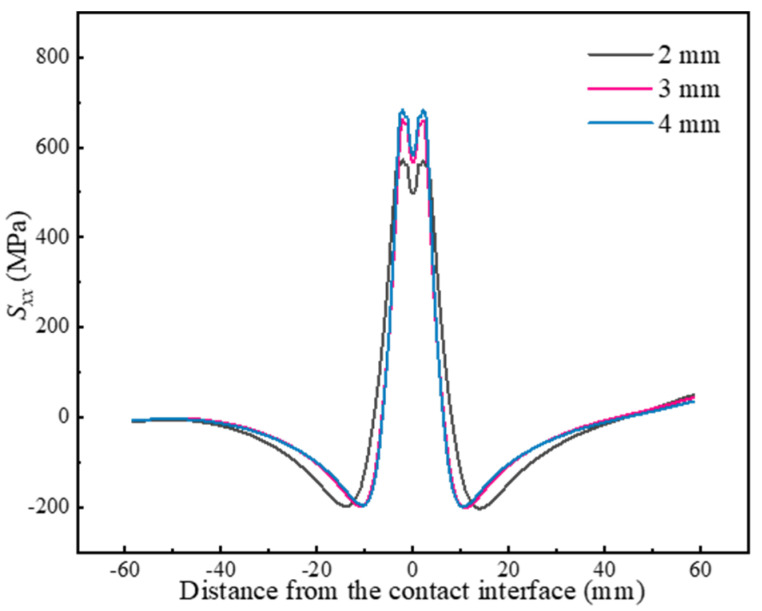
Effect of the amplitude on residual stresses.

The effect of welding parameters on residual stresses is further investigated, focusing on the *S_xx_* along Path 1. Figure 7 presents the residual stresses at different amplitudes. It is evident that the peak tensile stress increases significantly from 572 MPa to 683 MPa with increasing amplitude, while the peak compressive stress remains relatively unchanged. Additionally, increasing the amplitude from 2 mm to 4 mm results in a steeper residual stress distribution, with a reduction in the range of residual tensile stresses from 16 mm to 12 mm. Such a phenomenon, also noted by Bühr et al. [22], is likely attributable to the steeper thermal gradient induced by the increase in amplitude.

**Figure 8:**
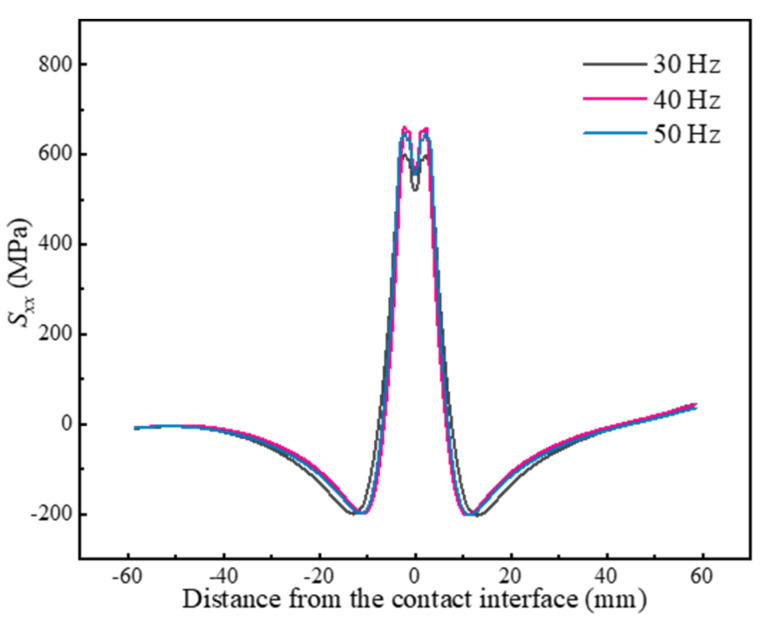
Effect of the frequency on residual stresses.

Frequency is also a key parameter influencing the heat input during LFW. The residual stresses at different frequencies are shown in Figure 8. As the frequency grows from 30 Hz to 40 Hz, the peak tensile stress significantly increases from 599 MPa to 661 MPa, and the residual stress distribution becomes more concentrated. However, the magnitude and distribution of residual stresses do not vary significantly as the frequency further increases. This may be due to the fact that the thermal gradient reaches a stable state when the frequency exceeds a certain threshold.

**Figure 9:**
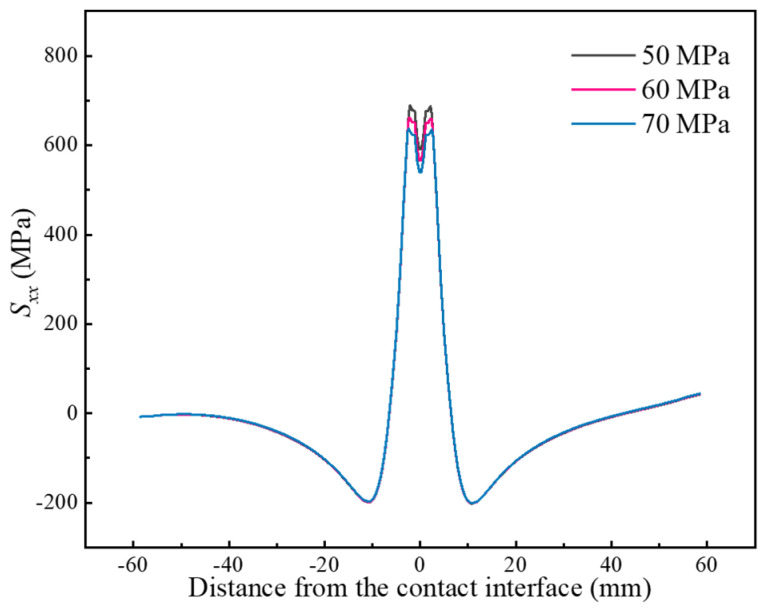
Effect of the forging force on residual stresses.

At last, the influence of forging force on residual stresses is also illustrated, as shown in Figure 9. It is evident that an increase in the forging force from 50 MPa to 70 MPa leads to a decrease in peak tensile stress from 688 MPa to 637 MPa. This trend is in agreement with the measurements of Romero et al. [20]. The reduction in peak tensile stress can be attributed to the balancing effect between the forging force and the tensile stresses near the contact interface caused by the cooling contraction. However, the range of residual tensile stresses does not exhibit significant changes with increasing forging force.

### 3.4. Prediction Method for Internal Residual Stresses

**Figure 10:**
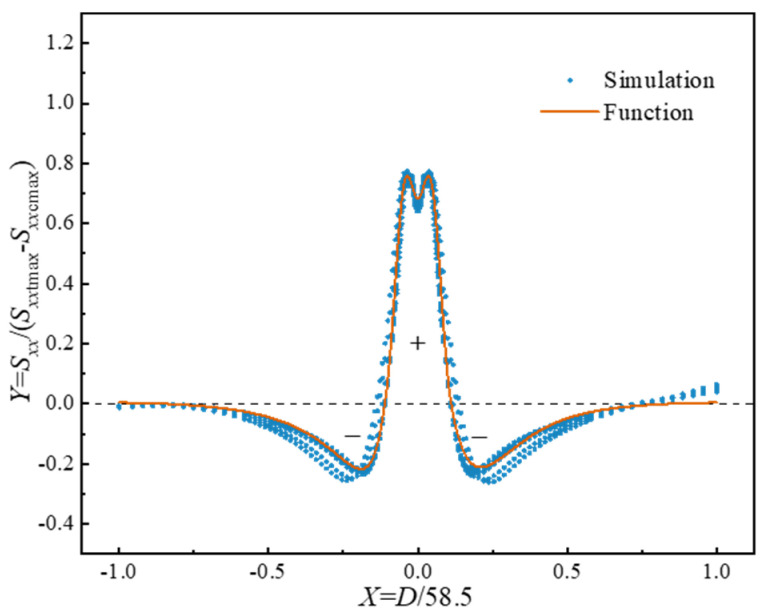
Curve shape of residual stress distribution.

According to the previous simulations, the internal residual stress distributions under various welding parameters exhibit similarity. Therefore, a dimensionless plot is established, as shown in Figure 10. In this plot, the vertical coordinate, marked as *Y*, is represented by *S_xx_*/(*S_xx_*_tmax_ − *S_xx_*_cmax_), and the horizontal coordinate, marked as *X*, is represented by *D*/58.5. *S_xx_*_tmax_ and *S_xx_*_cmax_ denote the peak tensile stress and peak compressive stress in the *x*-direction, respectively, and *D* represents the distance from the contact interface. Given the complexity of the stress distribution in LFWed Ti17, characterizing the overall residual stresses with a single function is challenging. Consequently, shape functions are developed separately for the tensile and compressive sections of the residual stresses. To begin with, based on the previous expression [13], an improved function that considers the bimodal distribution of tensile stresses is proposed as follows:

(4) $$Y_{\mathrm{tensile}}=A\left(X^{2}-0.0121\right)\left(1+BX^{2}+CX^{4}\right)e^{DX^{2}}+E.$$

The compressive stress distribution can be expressed using the following function:

(5) $$Y_{\mathrm{compressive}}=\left(\left|X\right|-0.11\right)\left(Fe^{G\left|X\right|}+He^{I\left|X\right|}\right)+E,$$

where *A*, *B*, *C*, *D*, *E*, *F*, *G*, *H*, and *I* are the fitting parameters of the shape function. A total of 1755 data points ensure that there are unique solutions for the fitting parameters. The fitting results are *A =* −55.76, *B* = 501, *C* = 253.6, *D* = −216.6, *E* = 0.0079, *F* = −17.85, *G* = −10.59, *H* = −0.8071, and *I* = −5.372. The coefficient of determination, *R*^2^, is 0.9848 and the root mean square error, RMSE, is 0.0312, indicating an excellent fit. The term (*Fe^G^*^∣*X*∣^ + *He^I^*^∣*X*∣^) ensures that the residual stresses decay to 0 at the edges. The continuity of the shape function is guaranteed by the terms (*X*^2^ − 0.0121) and (∣*X*∣ − 0.11). Due to the self-equilibrium requirement of the residual stresses, both tensile and compressive stresses must satisfy the balance of forces and moments [7]. The term *E* included in both shape functions makes the force balance satisfied. Additionally, the moment is balanced due to the symmetry of the shape functions.

**Figure 11:**
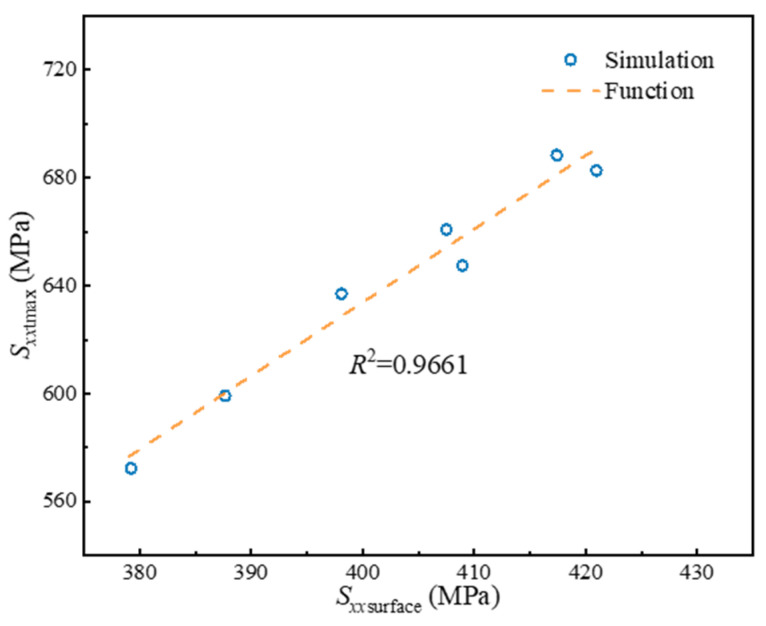
Relationship between internal residual stresses and surface residual stresses.

In order to predict the internal residual stresses in LFWed Ti17 by shape functions, it is necessary to acquire *S_xx_*_tmax_ and *S_xx_*_cmax_. Across various welding parameters, the *S_xx_*_cmax_ remains approximately constant at ~200 MPa, while the *S_xx_*_tmax_ can be indirectly inferred from surface residual stresses. According to Figure 11, the relationship between *S_xx_*_tmax_ and peak surface residual stress (*S_xx_*_surface_) can be established as follows:

(6) $$S_{xx\mathrm{tmax}}=2.72\times S_{xx\mathrm{surface}}-454.12$$

**Figure 12:**
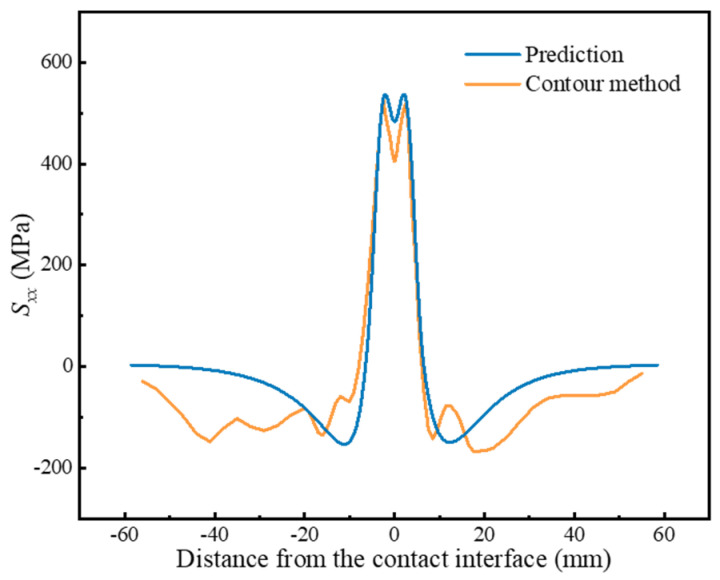
Internal residual stress distribution calculated using the prediction method.

To validate the accuracy of the prediction method, the *S_xx_*_surface_ measured using the X-ray diffraction method was introduced into Equations (4)–(6) to calculate the internal residual stresses, as shown in Figure 12. The internal residual stresses obtained from the prediction align well with the results of the contour method, which illustrates the accuracy of the prediction method.

## 4. Conclusions

In this work, the LFW is divided into two stages of oscillatory and forging to establish numerical models, respectively, considering the effect of flash generation on the temperature field. The numerical models effectively predict the residual stresses in LFWed Ti17. The tensile residual stresses, concentrated near the contact interface, exhibit the maximum in the oscillatory direction. Residual stresses in the oscillatory direction exhibit a bimodal distribution, with the peak (~661 MPa) located at 2 mm from the contact interface, and the valley (~566 MPa) located at the contact interface.

The evolution of stresses is related to not only inhomogeneous thermal expansion and contraction, but also the forging force applied. The variation of stress distribution in LFWed Ti17 occurs mainly within the duration of the applied forging force. With the increase in amplitude and frequency, the peak tensile stress increases with a narrower range of residual tensile stresses. Conversely, increasing the forging force only reduces the peak tensile stress without affecting the stress distribution.

Based on the shape functions describing internal residual stress distributions and the relationship between peak surface stress and peak internal stress, a prediction method for internal residual stresses in LFWed Ti17 has been developed. The internal residual stresses can be accurately and conveniently predicted by the measurement of surface stresses.

## Figures and Tables

**Table 1:** Chemical composition of Ti17 (wt%).

Element	Al	Sn	Zr	Mo	Cr	Fe	Ti
Content	4.97	1.97	2.01	4.09	4.00	0.029	Bal.

## Data Availability

The original contributions presented in the study are included in the article, further inquiries can be directed to the corresponding authors.

## References

[1] Boyer R.R. (1996). An overview on the use of titanium in the aerospace industry. Mater. Sci. Eng. A.

[2] Saresh N., Pillai M.G., Mathew J. (2007). Investigations into the effects of electron beam welding on thick Ti-6Al-4V titanium alloy. J. Mater. Process. Technol..

[3] McAndrew A.R., Colegrove P.A., Bühr C., Flipo B.C.D., Vairis A. (2018). A literature review of Ti-6Al-4V linear friction welding. Prog. Mater. Sci..

[4] Vairis A., Frost M. (1998). High frequency linear friction welding of a titanium alloy. Wear.

[5] Bhamji I., Preuss M., Threadgill P.L., Addison A.C. (2011). Solid state joining of metals by linear friction welding: A literature review. Mater. Sci. Technol..

[6] Turner R., Ward R.M., March R., Reed R.C. (2012). The magnitude and origin of residual stress in Ti-6Al-4V linear friction welds: An investigation by validated. Metall. Mater. Trans. B.

[7] Schajer G.S. (2013). Practical Residual Stress Measurement Methods.

[8] Rossini N.S., Dassisti M., Benyounis K.Y., Olabi A.G. (2012). Methods of measuring residual stresses in components. Mater. Des..

[9] Nikiforov R., Medvedev A., Tarasenko E., Vairis A. (2015). Numerical simulation of residual stresses in linear friction welded joints. J. Eng. Sci. Technol. Rev..

[10] Fu Y., Li W.Y., Yang X.W., Ma T.J., Vairis A. (2016). The effects of forging pressure and temperature field on residual stresses in linear friction welded Ti6Al4V joints. Adv. Manuf..

[11] Bühr C., Colegrove P.A., McAndrew A.R. An efficient numerical modelling approach to predict residual stresses in Ti-6Al-4V linear friction welds. Proceedings of the 10th International Conference on Trend in Welding Research & 10th International Welding Symposium of Japan Welding Society (9WS).

[12] Frankel P., Preuss M., Steuwer A., Withers P.J., Bray S. (2009). Comparison of residual stresses in Ti-6Al-4V and Ti-6Al-2Sn-4Zr-2Mo linear friction welds. Mater. Sci. Technol..

[13] Song X., Xie M., Hofmann F., Jun T.S., Connolley T., Reinhard C., Atwood R.C., Connor L., Drakopoulos M., Harding S. (2013). Residual stresses in Linear Friction Welding of aluminium alloys. Mater. Des..

[14] Xie P., Zhao H., Wu B., Gong S. (2015). Evaluation of residual stresses relaxation by post weld heat treatment using contour method and X-ray diffraction method. Exp. Mech..

[15] Daymond M.R., Bonner N.W. (2003). Measurement of strain in a titanium linear friction weld by neutron diffraction. Phys. B: Condens. Matter.

[16] (2020). Standard Test Method for Determining Residual Stresses by the Hole-Drilling Strain-Gage Method.

[17] Liu Z.Y., Dong C.Y. (2016). Automatic coupling of ABAQUS and a boundary element code for dynamic elastoplastic problems. Eng. Anal. Bound. Elem..

[18] McAndrew A.R., Colegrove P.A., Flipo B.C.D., Bühr C. (2017). 3D modelling of Ti-6Al-4V linear friction welds. Sci. Technol. Weld. Join..

[19] Zhao P.K., Fu L., Zhong D.C. (2014). Numerical simulation of transient temperature and axial deformation during linear friction welding between TC11 and TC17 titanium alloys. Comput. Mater. Sci..

[20] Romero J., Attallah M.M., Preuss M., Karadge M., Bray S.E. (2009). Effect of the forging pressure on the microstructure and residual stress development in Ti–6Al–4V linear friction welds. Acta Mater..

[21] Yan G., Crivoi A., Sun Y.J., Maharjan N., Song X., Li F. (2018). An Arrhenius equation-based model to predict the residual stress relief of post weld heat treatment of Ti-6Al-4V plate. J. Manuf. Process..

[22] Bühr C., Ahmad B., Colegrove P.A., McAndrew A.R., Guo H., Zhang X. (2018). Prediction of residual stress within linear friction welds using a computationally efficient modelling approach. Mater. Des..

